# P-1434. Revaccination of Pediatric Hematopoietic Cell Transplant Patients

**DOI:** 10.1093/ofid/ofaf695.1621

**Published:** 2026-01-11

**Authors:** Abbie L Blunier, Theresa Madigan, James Gaensbauer, Elizabeth H Ristagno, Kylie Juenger, Alexis Kuhn, Mira Kohorst, Annette Dauner-Olson, Laura Dinnes

**Affiliations:** Mayo Clinic Rochester, Rochester, MN; Mayo Clinic, Rochester, MN; Mayo Clinic, Rochester, MN; Mayo Clinic, Rochester, MN; Mayo Clinic Austin/Albert Lea, Austin, Minnesota; Mayo Clinic Rochester, Rochester, MN; Mayo Clinic Rochester, Rochester, MN; Mayo Clinic Rochester, Rochester, MN; Mayo Clinic, Rochester, MN

## Abstract

**Background:**

Revaccination is required for patients who receive a hematopoietic cell transplant (HCT) to reduce vaccine preventable illness. Revaccination requires an individualized plan due to differences in immunosuppressive therapy, immune reconstitution, age, and other challenges such as graft versus host disease. A structured approach is required to ensure prompt initiation of re-vaccinations and completion of the re-vaccination schedule. This study sought to evaluate the re-vaccination process at our center as a baseline for quality improvement.

**Figure:**
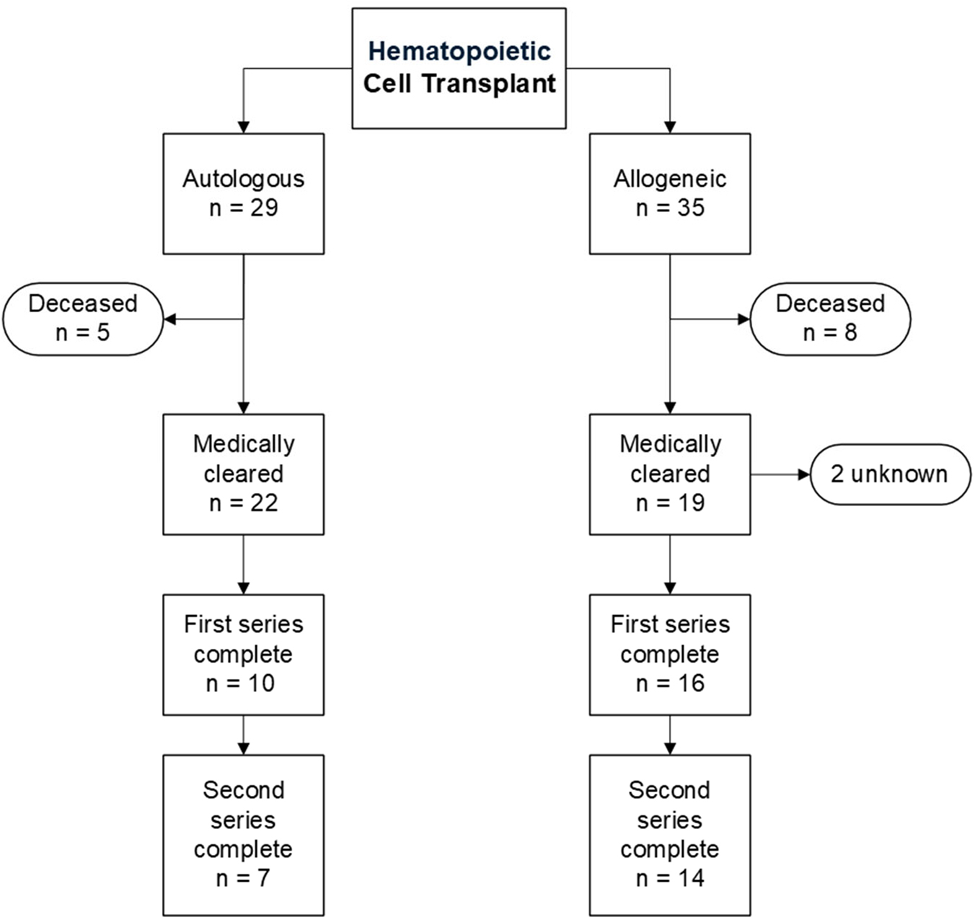

**Methods:**

We conducted a retrospective chart review of pediatric patients who received an autologous or allogeneic HCT between January 2019 and March 2024 to assess time to starting re-vaccination after post-HCT medical clearance and percentage of patients up to date within each series of vaccinations per internal protocol.

**Results:**

A total of 64 patients were evaluated, of whom 23 were female and 35 underwent allogeneic stem cell transplant. For analysis, 13 were excluded due to death prior to clearance for vaccination. Among those included, 42 patients were cleared for restarting vaccines, and 37 (88%) of those have received at least one vaccine, while only 25 (59%) have completed the first series of recommended vaccines.

The average time from transplant to administration of the first vaccine was 431 days for autologous HCT and 459 days for allogeneic HCT. The average time from medical clearance to the first vaccine was 66 days. The mean interval between the first and second vaccine doses was 97 days.

**Conclusion:**

The majority of eligible patients initiated re-vaccination after transplant, but rates for the receipt of full recommended initial series remained suboptimal, with notable delays with notable delays between transplant and the day of clearance as well as clearance and the first vaccine administered. These findings highlight significant gaps in post-transplant immunization practices and underscore the need for targeted interventions to optimize protection for this vulnerable population.

**Disclosures:**

All Authors: No reported disclosures

